# Social representations of animal diseases: anthropological approaches to pathogens crossing species barriers

**DOI:** 10.1051/parasite/2021032

**Published:** 2021-04-09

**Authors:** Frédéric Keck, Nicolas Lainé, Arnaud Morvan, Sandrine Ruhlmann

**Affiliations:** 1 UMR 7130 – LAS – CNRS/EHESS/Collège de France 52 rue Cardinal Lemoine 75005 Paris France; 2 UMR 208 – IRD/MNHN – “Patrimoines Locaux Environnement et Globalisation”, Muséum National d’Histoire Naturelle, Département Homme et Environnement 57 rue Cuvier CP 51 75231 Paris Cedex 05 France; 3 CNRS/EHESS, UMR 8173 54 boulevard Raspail 75006 Paris France

**Keywords:** Zoonoses, Biosecurity, Local knowledge, Cultural heritage, Mongolia, Laos, Australia

## Abstract

Debates about emerging infectious diseases often oppose natural conceptions of zoonotic reservoirs with cultural practices bringing humans into contact with animals. This article compares the representations of cross-species pathogens at ontological levels below the opposition between nature and culture. It describes the perceptions of distinctions between interiority and physicality, between wild and domestic, and between sick and dead in three different contexts where human societies manage animal diseases: Australia, Laos and Mongolia. Our article also argues that zoonotic pathogens are one of the entities mobilized by local knowledge to attenuate troubles in ordinary relations with animals, and shows that the conservation of cultural heritage is a tool of mitigation for infectious diseases emerging in animal reservoirs.

## Abstract (fr)

Les discussions sur les maladies infectieuses émergentes opposent souvent les conceptions naturelles des réservoirs de zoonoses et les pratiques culturelles qui rapprochent les humains des animaux. Cet article compare les représentations des pathogènes qui franchissent les barrières d’espèces à des niveaux ontologiques sous-jacents à l’opposition entre nature et culture. Il décrit les perceptions des distinctions entre intériorité et extériorité, entre sauvage et domestique, entre maladie et mort dans trois contextes où les sociétés humaines gèrent des maladies animales : l’Australie, le Laos et la Mongolie. Il montre également que les pathogènes zoonotiques apparaissent au milieu d’autres entités pour atténuer des troubles dans leurs relations ordinaires avec les animaux, et souligne que la conservation du patrimoine culturel peut être un outil de contrôle des maladies infectieuses qui émergent dans les réservoirs animaux.

## Introduction

It is estimated that 70% of new pathogens in humans are zoonotic, i.e., transmitted from animals to humans [8, 22]. The current COVID-19 pandemic, probably caused by a coronavirus endemic in bats, shows the high cost of public health measures for emerging pathogens and the need for their early detection in animal reservoirs [61, 62]. A “One Health approach” connects the surveillance of diseases in humans, animals, and their environment to understand how infectious diseases emerge and are transmitted from one species to the other, as well as within each species. However, the One Health approach has suffered from a colonial view of animals and their environments as resources to valorize, and failed to take into account non-Western views of animal agency [29]. Parasitologists, epidemiologists and ecologists have turned toward social anthropologists to understand the social and cultural factors allowing pathogens to cross the barriers between species and involve local populations in the management of animal diseases before they spread to humans [15].

The social anthropology of zoonoses has worked with the history of medical sciences to understand how concepts of emergence and spillover have changed the views of enzootic co-evolutions between humans, animals and parasites [33]. Hotspots of zoonotic emergence have become sites of investigation on the multiple relations between humans and animals [5]. Concepts of proximity and contact, often used in One Health representations of zoonotic transmission, have been problematized using materials from narratives on broader multi-species ecologies and histories [1, 41]. The urgency of biosecurity interventions, focusing on early detection using sentinel devices, is often contrasted with cultural practices which need to be regulated, such as bushmeat hunting or wildlife markets [23, 30]. However, social anthropology cannot oppose a global view of nature as self-regulating with its cultural localizations producing risks of emergence. The opposition between traditional societies fearing dangers and modern societies calculating risks appears less relevant in a world where common uncertainties require a plurality of cognitive operations [11, 21, 32].

As the term “culture” is not adequate to study the perception of zoonotic risks, it is more fruitful to focus on representations of species barriers. In all societies, humans make inferences about the behaviors of animals based on their similarities and differences. A zoonotic disease is an opportunity to question what humans and animals have in common based on shared representations, which stabilize the potential crisis opened by the disease. The term “ontology” has been used in the debate within anthropology and science studies to overcome the opposition between nature and culture, since it takes seriously the statements about what “is” and what “is not” in a collective form of life, particularly in the perception of invisible entities such as spirits, ghosts or microbes [19, 60]. However, the term “representation” is more open to investigation on how these statements are stabilized over time, inscribed in modes of production and valorized by institutions. Species barriers are problematic sites not only for disease management but also for ethical reflexivity and social justice: they raise the question “What made me sick?” in a web of vulnerable entanglements between humans and non-humans. While the concept of cultural behavior focuses on human-animal proximity as an obstacle to the global management of zoonoses, the concept of social representation helps to include societies living with animals in global health [13]. When domestic and/or wild animals are killed to prevent the spread of their diseases to other animals or to humans, this causes ontological and ethical problems because humans consider animals not only as commodities that can be destroyed in a food chain but also, in some cases, as companions within kinship relations of attention and care [16, 17]. A sick animal – domestic, wild, or pet – is an ambivalent being for humans who have to protect it against the disease but also protect themselves from the disease; hence the need to compare different ways to mitigate this existential tension through collective representations.

## Method

Philippe Descola [11] has argued that four ontologies are available for the human mind to compare human and non-human beings based on the polarities of inference about agentivity: naturalism (different interiority, same physicality), animism (same interiority, different physicality), analogism (different interiority, different physicality), totemism (same interiority, same physicality). For Descola, while animism is more developed in Amazonia and Siberia, analogism in Africa, central America and Asia, and totemism in Australia, they can also coexist in the same situation, depending on the relations between humans and animals at stake (hunting, breeding, labor, etc).

We took this hypothesis as a starting point for empirical comparison. We did not seek to illustrate Descola’s ontologies in our fieldwork, but we took them as a stimulating hypothesis to raise questions on the social representations of species barriers. Rather than start from global health measures to search how they can be applied in different locations, which supposes an opposition between interventions on nature and cultural obstacles [9], we have started with animal diseases that cross species barriers differently in various ontologies: totemism on Australia, analogism in Laos, and animism in Mongolia. We kept in mind that these ontologies coexist with other ontologies in each of these three societies and found results that confirm these hybridizations.

Frédéric Keck has supervised the collective construction of a questionnaire on pathogens crossing species barriers, based on his own research in south China [24]. Three anthropologists used this questionnaire in societies they had studied before through other topics of research. Arnaud Morvan studied Australian indigenous art in relation to colonial history, relationship to the land, totemic places, and species. Nicolas Lainé studies human–animal labor and local knowledge on elephants including domestication and ethnoveterinary practices in post-colonial Laos and India. Sandrine Ruhlmann studies techniques and material culture mobilized by food practices in post-communist Mongolia.

First results have been surprising because some of the questions took different meanings in the social contexts where anthropologists asked them and were often confused with veterinarian experts. From a quantitative point of view, the question “Can diseases be transmitted from animals to humans?” received a positive answer among 60% of the people we interviewed in Australia, 0% in Laos, and 85% in Mongolia. When we asked our informants about their understanding of bacteria or viruses, we often had very vague answers, or simple repetitions of public health campaigns. However, we used ontological questions as ways to describe ethnographically the parameters of social representations of species barriers, and adapted them to local conditions. Rather than ask if our informants knew about a zoonosis, we asked them how they represented microbes and how microbes crossed species barriers causing pathologies among animals and humans. While the knowledge of microbes as “natural” entities was very unstable, our interviews aimed at tracing the map of entities that were mobilized to cope with a potential disease spreading through animals. The three researchers thus combined the qualitative questionnaire method, open-ended discussion, and participant observation by taking part in rituals involving animals, animal labor or herding practices.

*In Australia*, Arnaud Morvan investigated Aboriginal representations of flying foxes (*Pteropus* spp.) as natural hosts of the Hendra Virus (HeV) on the tropical coast of North Queensland. Since 1994, this virus from the Paramyxoviridae family has killed 89 horses and 4 humans in Australia, spreading from flying foxes to horses and from horses to humans. HeV has been found in the four species of Australian Pteropus: *Pteropus alecto, P. scapulatus, P. conspicillatus*, and *P. poliocephalus* [51, 57]. Biosecurity interventions have aimed at controlling the virus in the animal reservoir, particularly by separating horses and flying foxes. Despite their long-term relations with flying foxes, indigenous groups have rarely been involved in the management of the bat populations, either in bat shelters or in natural parks. While Aboriginal people have used flying foxes as bush meat for centuries without noticeable human transmission of HeV, and prepared them as a traditional medicine to treat respiratory diseases, the subject has never been investigated and public health experts have ignored or discouraged these medicinal practices and associated knowledge.

*In Laos*, Nicolas Lainé studied the representations of tuberculosis (TB) by elephant owners and mahouts (elephant handlers). During the past 20 years, the growing number of captive elephants tested positive for TB in various institutions worldwide (in western zoos or in some tourist camps in Asian elephant range countries) has caused public health concerns, mainly related to the significant risk of propagation. Globally, TB remains the second leading cause of human death after HIV, generating nearly 1.5 million deaths annually. In this context, the concern with elephant TB has led to unprecedented numbers of seroprevalence and epidemiological studies to help prevent and treat the animals [35]. Southeast Asia hosts more than 15 000 elephants in captivity and has the highest prevalence of tuberculosis in the world. A survey made in Laos [31] indicated a prevalence of 34% among elephants tested, the highest rate for TB in elephants in Asia so far. Since TB is a slow endemic disease, and is not necessarily the primary cause of death for infected patients, there has been no public health crisis after this warning. However, because TB in elephants is considered a reverse zoonosis transmitted from humans to animals, surveillance aims at protecting humans and animals [26].

*In Mongolia*, a post-communist country with 70.9 million head of domestic animals (sheep, goats, cattle, horses, and camels), and 3.2 million inhabitants, and where one third of economic production comes from herding, Sandrine Ruhlmann conducted ethnographic research in three provinces (Töv, Hentij, and Gov’sümber) during the winters and summers of 2014–2019. In these areas, families practice extensive, polyspecific, and nomadic herding, according to predefined routes across seasonal rotating pastures to preserve soil and water resources. Sandrine Ruhlmann has focused on the management and surveillance of three animal diseases that were controlled under the Communist government, but that have since chronically re-emerged, developed, and spread in different ways over the vast Mongolian territory: brucellosis (a bacterial disease – *Brucella melitensis* –, endemic and widespread, zoonotic), anthrax (a bacterial disease – *Bacillus anthracis* –, localized, zoonotic), and foot-and-mouth disease (FMD) (a viral disease – Picornaviridae *aphthovirus* –, localized and pandemic, non-zoonotic) [48, 49]. The prevalence of brucellosis is high throughout the entire territory, among human and domestic animal populations (mainly ruminants), with disparities between the 21 provinces, depending on whether NGOs have implemented a vaccination program for several years. An important part of the rural population (sometimes up to 11%) is infected, especially animal workers [2]. However, interviewed herders do not fear the “bacterium” (*bakteri*) as they know that “brucellosis” (*brucellëz*) is transmitted through contact with milked milk, consumption of dairy products and undercooked animal flesh, saliva, and blood at the time of delivery, but are not aware of the correlation between brucellosis and abortion in animal and human populations. By contrast, they are afraid of “anthrax” (*boom*), because they know that the “bacterium” (*bakteri*) is highly contagious, transmitted by hair, wool, hides, bone, air, meat, and contact, and is fatal for animals and humans, and can be widespread over the whole territory, excluding southern desert provinces. Finally, “foot-and-mouth disease” (*šülhij* or *šülhij övčin*) is the most familiar disease because it is “*highly visible*” in the media and “*highly contagious among animals*” (air, contact with bodily excretions such as feces, urine, milk and saliva from affected animals), and has chronically emerged among domestic and wild bovine, ovine, and caprine animals, which are in contact during seasonal migrations, in the four eastern provinces. Herders do not fear this “virus” (*virus*) in the sense that it kills only animals, domestic and wild, but they fear that their herd may be infected and that, according to international and national regulations, they must slaughter all or part of their herd, their only resource and source of income [12, 46].

**Table:** 

Animal Diseases	Hendra Virus	Tuberculosis	Brucellosis, anthrax, Foot-and-mouth disease
Animal Species	Bats	Elephants	Sheep, goats, cattle, horses, camels (domestic); and gazelles (wild)
Locations	Australia (Queensland)	Laos	Mongolia
Predominant ontology	Totemism	Analogism	Animism
Fieldworker and specialities	Arnaud Morvan (art, totems and rituals)	Nicolas Lainé (animal labor, local knowledge)	Sandrine Ruhlmann (food practices and techniques)

To analyze the representations of zoonoses, we supposed that indigenous populations in Australia, Laos, and Mongolia engage different polarities of human experience in contrasting ways. We built the interviews around the following polarities:

- Interior/exterior: Do pathogens come from inside or outside the animals’ body? What are the conceptions of transmission by contact? What are the proper relations of proximity and distance between humans and animals?
- Wild/domestic: How does domestication change the management of animal diseases? Are wild animals treated separately from domestic animals? How is the separation between wild and domestic traced in relation to the habitat?
- Sick/dead, versus culled/slaughtered: How are sick animals treated in comparison with dead animals? Are sick animals eaten, buried or burnt? What is the difference between animals that died from zoonoses and animals that died from other diseases?

## Results

### a) Interiority/externality

The notion of interiority describes, in various societies, ideas of souls, breath, energy, and forces that animate the body and leave it at death. Physicality describes substances such as blood, flesh and bones that compose the body and are fragmented at death. The treatment of disease involves knowledge on interiorities and physicalities shared (or not) between humans and animals.

#### Australia

In Far North Queensland, flying foxes (*kambi*) are considered bush meat (*minja kambi*), traditional medicine, and totemic referents [36, 45]. Today, pathogens such as the Hendra virus are sometimes associated with flying foxes, but this does not prevent people from consuming them, either cooked in a ground oven or in soup. Most people are aware of the potential presence of the virus and special attention is taken during the cooking process to “kill the germs”. While this practice of long and slow cooking is considered traditional, it coincides with non-indigenous public health messages about pathogen transmission, imported into local discourse. Early ethnographer Walter Roth [47] mentioned the alimentary use of bats amongst the North Queensland indigenous people at the end of the 19th century, but no medicinal practices have ever been documented. Soup made from flying fox is also considered traditional medicine to treat certain types of respiratory problems, less severe but analogous to the respiratory syndromes caused by the Hendra virus. It remains to be seen how this practice is connected to hypotheses on immune properties in bat organisms that make them healthy carriers of pathogens [57].

However, for indigenous people, pathogens do not only come from inside the animal but rather from outside as they are connected with totemic sites and associated species. In Australia, totems are prototypal hybrid beings that embody indigenous concepts of interspecies connections, materialized in specific classes of humans and non-humans sharing a common mythical ancestry and common attributes. They are often attached to a specific animal species but not exclusively; they can also refer to plants, minerals, elements, psychological traits, or sometimes diseases [14, 56]. People are usually associated with several totems (individual conception totems, clan totems, sexual totems, kinship totem), and inherit certain rights and responsibilities toward a multiplicity of animal species (or element, plants, minerals, etc.) and their associated place in the landscape, often considered sacred [39, 40]. A person cannot eat his/her own totem. As a Yalanji elder explains:

“[this person] would get sick. He wouldn’t die but he would get sick, sore guts, stomach pain you know. Then you know it’s wrong” (Interview, 2015).

Cases of disease totems are rare but highly significant, especially in their relation to animal species. Early ethnographers Baldwin Spencer and Francis Gillen [52] recorded an Arrernte (central desert language group) totemic story linking an animal species, a place and an infection related to a narrative and sickness called *erkincha*, a disfiguring disease (*Treponema* bacteria) associated with syphilis. Part of the *erkincha* mythical story is concerned with Alchipa, a totemic being, related to a small desert marsupial (western quoll, *Dasyurus geoffroii*), that carried the disease *erkincha* and disappeared after creating several landmarks. Since then, the Alchipa has been associated with a rock called “Aperta atnumbria,” a specific site that causes diseased growth (early stage of syphilis). Spencer and Gillen also observed a restriction towards eating the marsupial species (*Dasyurus geoffroii*) called after their Alchipa ancestor, to prevent the disease. The existence of food restrictions applied to animal species carrying a disease clearly confirms the existence of specific local knowledge about disease transmission between animals and humans [58].

Considering diseases in relation to the connection of an animal species (*Dasyurus geoffroii*) a “place” (“Aperta atnumbria” rock) means that pathogens are not fully independent agents but rather a web of interspecies and ecological relations. In the Australian indigenous conception, the shared totemic origin of people and animals theoretically allows vital energies as well as pathogenic agents to circulate between human and non-human species. The pharmaceutical value of bats remains to be thoroughly researched but their medicinal use has been observed in the entire Asia–Pacific region and beyond [44]. The totemic conception reverses the image of flying foxes from a potentially pathogenic to a potentially therapeutic species.

#### Laos

Tuberculosis has revealed the conceptions of interiority and exteriority engaged in elephant labor and care. Laos is known as “the country of one million elephants” (*Lan Xang*), notably because elephants have been raised by kings for transportation and by colonial powers for the logging industry, and now by environmental associations who organize ecological tours. Over these different periods, mahouts have developed knowledge to work with elephants and to care for their health using local plants. But for them, the presence of external signs on elephant bodies (such as runny nose) does not appear relevant. According to some mahouts, after 4 or 5 days working in the forest, the elephant’s back becomes emaciated, and some even change their skin colour in the forest. However, all mahouts say that once their task is completed, and when they let their elephants wander freely in the forest, it only takes a few days to recover their ideal weight.

By contrast, the perception of internal signs of health is expressed in the language of spirits. Like all big animals, elephants are believed to be inhabited by *phi* (spirits), and like humans they are animated by a vital force, the *kwaan*. Every night when mahouts leave their elephant in the forest after a day’s work, they invoke the spirit of the forest (*phi pa*) and the spirit of soil and earth of a specific territory (*chao chao gift dee*), and ask them to protect the elephant in case of attack by other animals – like snakes – but also by evil spirits (*phi phai*).

This ritualization requires the intervention of a specialist. The *mo xang* firstly intervenes during the training of the animal, and then when an elephant becomes weak and does not respond to his mahout, or in case of attack by a bad spirit. Through incantation and magical words (*khata*), he can cure the elephant and chase away bad spirits. Moreover, every year, the shaman performs the annual *baci* ceremony offered to village elephants. This ritual aims at gathering all the vital forces (*kwaan*) within elephants’ bodies, in a form of rejuvenation, but it is also offered occasionally to thank the animals for the work they carry out for humans.

In villages, another specialist intervenes to ensure the well-being and health of elephants using medicinal plants to cure humans as well as animals: the *mo ya*. Their manuscripts of plant compositions do not mention respiratory disease like tuberculosis. Even though humans, elephants and other domestic animals such as buffaloes are vulnerable to spirits (*phi*), considering tuberculosis (*wan nga lok*) a general disease (*panyaat*) crossing the interspecies barrier is hardly thinkable.

#### Mongolia

Livestock health is highly meaningful for interviewed nomadic herders. It represents what a household possesses: a “*healthy and prosperous herd is an indicator of happiness*”, meaning that “*everything has been done in a good way*”. To prevent misfortune, people are expected to act properly in everyday life or at specific events where the fate of the soul is at stake, i.e. the rebirth of the deceased’s soul or the fixation of the new-born child’s soul in his/her skeleton. A sick animal, as much as a sick human, means that “*something has been done in the wrong manner that makes the master spirits of nature or Buddhist divinities angry, or that attracts wandering souls of the dead*”. Households have “*something to repair*”, “*happiness to (re)call*”, by themselves and/or by bringing a monk, a shaman, and a veterinarian, one after the other, without any hierarchy or distinction. The consultation of a ritual specialist leads Mongolian herders to list a set of spiritual entities on which their happiness and health depends: nature spirits-masters, Buddhist divinities, souls of the dead to be reborn, and souls of the dead condemned to wander.

Humans and animals are considered similar as far as their bodies are made of flesh and bone, but different with regard to other components: if both have a “vital force” (*süld*), a kind of energy that irrigates organs and sustains life, and a “breath of life” (*am’*), that outlives skeleton at death, only humans have a “soul” (*süns*), a unit of life residing in their skeleton and animating their body. Meritorious actions practiced during life allow their “soul” (*süns*) “to be reborn” in the body of another human. Herders cannot conceive the rebirth of a human soul in the body of an animal (as in the Buddhist doctrine) because such a conception would mean a breach within unity and perpetuation of the human species [50].

In addition to more recently imposed western medicine, there are at least two medicines coexisting among Mongolian people: popular medicine closely related to shamanic healing practices (*dom*), and high medicine derived from the Tibetan tradition. These healing practices can act on different diseases, such as anthrax, rabies, FMD, and scabies [20]. Physical treatments (*em*) are not restricted to diseases, and magical-religious therapies (*dom*) to misfortunes. Both types of treatments involve the same technical gestures: press, wrap, wash (with a damp cloth), pierce (with a large needle), incise (with the knife blade), scrub, cauterize (with a mud or tea poultice, with something hot), burn, fumigate (with incense), sprinkle (libation of milk), swallow (a curative substance, salt, meat soup, sulphur powder, extract of willow bark or tobacco, blood of horse or gazelle, raw milk), scare (wandering souls of dead), and offer food to divinities. These actions simultaneously aim to remove angry spirits of nature or wandering souls of the dead and cure the disease [6, 20, 49].

This dual conception of the human being – body and soul – has implications in the representation of the emergence and transmission of pathogens. Most herders know they should not enter into contact with substances of animals sick with brucellosis or anthrax: saliva, urine, excrements, fetal water, blood, raw flesh and milk, etc. However, most of the time, local know-how involves manipulating these substances, such as pus out of the internal (organs, flesh), or external body parts (skin, hair, nails). The verb *garah*, “come out”, qualifies a disease outbreak or emergence: it comes from the soil, it is transported by the Earth or air, and at the same time it comes from infected animals (internal and/or external part of their body) and from the anger of different spiritual entities. Some pathogens reside under the skin or inside the body (i.e. brucellosis, intestinal anthrax) and most of their symptoms are “*invisible*”, while other pathogens are external, “*visible*”, located on the skin (cutaneous anthrax, FMD), and on the eyes, nails, or mouth (FMD), etc. Surveillance consists in “*continuous attention to animals’ behaviour*”, meaning in particular “*their appetite, sleep, and movement, if they urinate and defecate normally*”, and if “*they isolate themselves from the rest of the herd*”, “*they refuse to go to pasture at dawn*”, “*they stay and lie down, go around in circles, or tremble*”, etc. Daily herding activities – carried out inside the camp – allow herders to detect something wrong because they are moments of proximity.

Zoonotic pathogens thus mobilize totemic conceptions of shared properties in ancestral places in Australia, notions of forces and spirits in Laos, and manipulation of substances in Mongolia. These contrast with the naturalistic opposition between animal bodies and human knowledge at the basis of pharmaceutical techniques.

### b) Wild/domestic

“Wild” usually defines spaces untouched by human interventions where pathogens silently mutate, and “domestic” spaces of cohabitation with humans where pathogens are amplified. Our investigations have blurred these oppositions.

#### Laos

Asian elephant (*Elephas maximus*) is a specific or limit case of domestication. Like other Tai-speaking populations across South and Southeast Asia, in Laos the Tai-Lue and Tai-Lao mark a strong difference between the world of the forest (*pa*) and the world of the village (*ban*). This differentiation is used to distinguish forest or wild elephants (*xang pa*) from village or domestic ones (*xang ban*). Historically in Asia, the renewal of village elephant herds involved forest captures and resulted in a specific form of domestication [27]. In Laos, captures and socialization involved elephant specialists (*mo xang*) who removed forest spirits from the elephant’s body and called domestic spirits to take care of the elephant’s health. This shows that the construction of a so-called “domestic” animal relies on the transformation of a “wild” one.

In villages, contrary to buffaloes or other livestock, elephants are believed to be part of the family. In most of the elephant owner households, photos of elephants are displayed along with all family members. Elephants are also protected by the spirit of the house (*phi huen*), which is not the case for buffaloes. For example, a mahout in Viengkeo villages said that for several days, he was not able to find his elephant in the forest, and finally managed to get the elephant back. He said that the spirit of the house had hidden the animal, depriving the family of incomes, because the wife and husband were arguing all the time. Finally, after promising the spirit that they will not argue anymore, he was able to find the elephant.

The recent emergence of elephant TB is probably the result of transmission from humans or cattle and not from interactions with wild congeners. Nevertheless, since village elephants are still in contact with their forest congeners, there is a risk of transmission of TB throughout the forest, including wild elephants and other species. But this risk does not appear to be relevant for the mahouts and elephants owners.

#### Australia

According to epidemiologic research, the Hendra virus is endemic in Australia among flying foxes, and was recently introduced in horses, and then jumped from horses to humans. Flying foxes live in forests, swamps, and coastal areas and have never been domesticated (despite some bat shelters built for temporary rescue by conservationists). Yet numerous colonies can be found in urban areas, including parks, gardens, or even isolated trees in city centres [55]. Most recent studies tend to show that there are more colonies in or around cities than in remote areas. City edges seem to be the main contact zone between bat and human activity, including horse grazing near the forest. For most non-indigenous Australians living near flying fox colonies, these animals represent an considerable source of public nuisance. People interviewed in the Cairns regions speak about the noise, smell, crop damage, and more recently, the Hendra virus. The words “flying rats” or “plague” come up during informal conversations.

Paradoxically, Hendra virus also brought to collective attention the fragility of some flying fox species (*Pteropus conspicillatus*) and led to protection policies. Bat shelters are places where it is possible to treat, cure, protect and in some cases domesticate flying foxes (once healed, animals unable to be reintroduced into the wild are kept in the refuge and given names). Ultimately, both strategies tend to reproduce and impose the naturalistic distinction between wild and domesticated animal life in tropical Australia and maintain a strict separation between humans and non-humans.

For indigenous communities, who often have flying fox colonies in or near their settlements, totems overlap the dichotomy between wild and domestic. Individuals can have very strong connections and sometimes close communications with their totemic animal species, even if they are considered wild or even dangerous. Flying foxes are highly regarded amongst Aboriginal people. There are several totemic sites associated with flying foxes in and around Yalanji country, and most of them are classified as restricted sacred areas. In the Australian totemic system, the elements inside the totemic geographical network are considered familiar and domestic entities. By contrast, what is outside this network, and does not have a totemic name, belongs to the virtual domain of the unknown. In this context, the flying fox, as a well-established totem, is closer to the domestic domain, while horses, as an introduced species, is closer to wilderness. The longstanding alimentary and medicinal use of the host species of Hendra virus tends to question the role of flying foxes as the origin of the zoonosis and to rethink cross-species transmission. From the perspective of long-term co-evolution in a network of kinship, the introduction of foreign domesticated animals (horses) to Australia has triggered a new human disease, born from the forced contact between domesticated and wild species. The wild, here, is not the reservoir species but the invasive species.

#### Mongolia

In late summer and early fall, Mongolian herders occasionally exit the camp to hunt wild animals far from the pastures. They hunt to eat their flesh and use their fur and leather. However, they sometimes hunt to protect their herds from predators (wolves) or from infected wild animals (wolves, foxes, and marmots, infected with rabies or the plague), i.e. when symptoms are detected and animals become ill or die. Although they are not in close or daily contact with wild animals, domestic animals have occasional contact with them: for reasons of predation and migration, some wild species move more often and farther than domestic animals whose extensive herding is partly based on the principle of human nomadization. Also, very recently, the Mongolian government has ordered hunters and the military to shoot herds of gazelles, wild animals believed to be responsible for the emergence and spread of the FMD virus [48, 49]. Mongolian herders admit different degrees of domestication within their livestock, notably regarding horses, a half-domestic half-wild species. Horses are ambivalent means of transportation, used by monks to send away the soul of a deceased in funerary contexts. Wild animals with fur or feathers allow wandering souls of the dead to approach the living and roam around their homes [50].

Uncertainties concerning the wild reservoir of animal diseases have caused problems at the government level. After the FMD outbreak of 2010, the lack of a livestock vaccination program led the government to shoot thousands of wild white-tailed gazelles (*Procapra gutturosa*), voluntarily and wrongly accused of being the pathogen reservoir [4, 10, 42]. The goal was to stop the re-emergence of FMD on the border between Mongolia, Russia, and Inner Mongolia, a territory under the authority of China. Another objective was to reply to the Chinese government who accused nomadic herding in order to encourage sedentary lifestyle in Mongolia. This governmental decision to shoot gazelles produced tensions between some conservationists who consider gazelles a protected species – in accordance with the government decree –, herders who legally but seasonally and restrictively hunt them, and poachers who hunt them illegally and without restraint.

The herders pointed out a contradiction in the government’s strategy: it claims that the virus “*doesn’t cross natural barriers (mountains, desert)*” and “*can’t spread from eastern to western provinces, reach the capital and put the whole country under quarantine*”, and at the same time it recognizes that the virus “*can cross state borders and create a pandemic*”. In 2010, when the government decided to cull thousands of gazelles to eradicate the FMD outbreak in response to pressure from neighboring states (China and Russia), experts and NGOs asked the government to respect the law of wildlife conservation. Herders said the Mongolian authorities preferred to lie to the population rather than confront and refute the Chinese government. While the frontier between humans and animals was strictly respected, particularly during the quarantine period, the separation between wild and domestic was more easily questioned by herders, because it resonated with other frontiers between political territories (Mongolia and China) and social groups (experts, herders and poachers).

While totemic conceptions in Australia reversed the naturalistic opposition between wild and domestic, practices of animal breeding in Laos and Mongolia anticipate them through spiritual and political distinctions.

### c) Sick/dead

Zoonoses are conceived as diseases potentially leading animals and humans to death. However, there is not a biological continuum from disease to death but a moral situation of trouble that requires precautionary measures. The carcasses of sick animals must be handled with care to avoid zoonotic transmission.

#### Australia

In Far North Queensland, flying foxes are harvested and killed by indigenous people as bush meat, using a stick or sometimes a stone or a rifle. They are never killed for purposes other than alimentary or medicinal, and no difference is made between healthy or infected bats, mostly because flying foxes are healthy carriers of HeV and therefore show no signs of sickness. However, flying foxes are carefully prepared in order to kill the pathogens, but the carcass is destroyed without any particular precaution after culinary or therapeutic preparation and consumption. Aboriginal people interviewed were opposed to the dispersal or culling of flying foxes to prevent potential HeV outbreaks.

Farmers and horse owners have a different approach to dealing with bats, which they do not see as a resource but as a problem, mainly for crops and for the threat they represent to horses and people. The high number of viruses they potentially carry is often brought up as the main reason to disperse or cull bats. Despite their protected species status, some farmers still do not hesitate to randomly and illegally shoot flying foxes encountered on their property. An application form issued by the Department of Environment and Heritage Protection in 2006 authorizes and regulates their shooting. The document indicates that “prior to disposal, dead flying-foxes must be held for 24 h and be available for inspection by an EHP or conservation officer”. In cases of Hendra outbreaks, most horse owners are generally in favor of bat culling or massive dispersal, which remain rare. Infected horses themselves are euthanized to prevent the virus spreading. Importantly, any horse that has been in contact with an infected horse is put into quarantine.

Today, in most cases, indirect action to push flying foxes away based on netting, sound, light or smoke, is still officially preferred. Dispersing a flying fox colony can, however, be stressful for the bats, especially the young, and often leads to a rise in the viral charge [25]. Destruction of bats’ traditional environments and dispersals are thought to be the main causes of Hendra virus diffusion [43].

#### Mongolia

In general, domestic animals are slaughtered in the camp and eaten in the house, and wild animals are shot and eaten out of the camp or house. Slaughter requires killing the animal without bloodshed on the ground, considered ancestor’s soil. The herder incises the animal down its sternum, sticks his right hand into the fat groin and presses the thoracic aorta to cut animal blood circulation and cause rapid death. He then cuts the aorta and lets the blood flow into the rib cage. The slaughterer and the killer are both required not to section any bones nor to cut the animal at the joints of the skeleton. This treatment allows for the consumption of meat of domestic and some wild animals (e.g. marmot meat).

When an FMD outbreak occurs, domestic animals are killed and the flesh of infected animals is not consumed, even though herders know that eating this infected meat will not make human consumers ill – the flesh of diseased animals is not eaten in general. If the disease is not contagious, the carcass is left beyond the camp boundaries and scavengers will eat it. Otherwise, the sick domestic animal undergoes another treatment: herders or veterinarians take away the infected animals (out of the camp, in the middle of the steppe, wild area), shoot them (cattle) or slice the throat (goat, sheep) thus letting blood flow onto the soil, push them into a deep hole – a mass grave –, disinfect carcasses with lime and cover them with soil. Before burying, they sometimes destroy them in a large fire. This means that contagious non-dismembered and bloodied carcasses are buried.

There are also differences and similarities between the treatment of sick animals and the funerary treatment of humans who died of old age or disease. Since the Communist period, humans are buried in coffins. Before placing the coffin with the body in the pit, members of the funerary cortege make libations to purify the pit, the grave and the coffin, i.e. by extension the body and the soul of the deceased. In both cases (deceased humans considered as polluting and contagious animals), the government makes large pits in anticipation. After the disinfection of the pit and the body, the family purifies the house and personal belongings of the deceased in the same way as, in the context of FMD contamination, herders purify enclosures, tools and remaining animals. They use consecrated incense fumigations and libations of consecrated water and milk mixture, in addition to the disinfectant (lemonic acid) provided by the authorities. Finally, before going back home, herders must purify internal and external parts of their bodies: they ingest a sugar cube dipped in a consecrated mixture of water and milk, and they rub their hands and face with a cotton ball soaked in the same mixture [48, 50].

#### Laos

The treatment of elephant carcasses differs highly from Australian and Mongolian contexts. Informants mentioned that elephants know when they are going to die, and they let them go alone into nearby forest areas. Therefore, elephants are not killed by humans even if they are seriously ill or dying. However, an owner was said to deliberately shoot his elephant, not because it was ill or sick, but because it was too dangerous for humans. More generally, when an elephant dies, the owner calls the ritual specialist who, by a new ceremony, literally separates the animal from the household and organizes the funeral in the same way as for humans. During this ritual, he addresses the elephant’s vital force (*kwaan*) and all the spirits to inform them that they are no longer part of the household, meaning that the animal no longer belongs to the elephant owner’s family. The same treatment is applied to human funerals before bodies are buried. And only once this separation is made, the owner decides if the body of the elephant is burned, buried or even consumed. In Laos, eating elephant flesh is considered as taboo just like in South Asia [54]. Nevertheless, some Tai-Lao informants mentioned that they sometimes eat elephant flesh. The only elephant body parts they collect after death are the tusks (for male elephants), and hair from the tip of the tail.

The treatment of animal carcasses is thus different from the treatment of human cadavers in our fieldwork, but this difference was not justified by the opposition between commodities and moral beings as in naturalistic societies.

## Discussion: the meanings of zoonoses for politics of heritage and conservation

Our ethnographic studies thus show that zoonoses engage fundamental aspects of social life. When pathogens cross species barriers, they challenge numerous binaries constitutive of the social order, of which we have chosen to investigate three: interior/exterior, wild/domestic, and sick/dead. We have found that people who live with animals often reverse positions of governmental experts and urban populations on these binary oppositions, because these people represent spirits who move from the interior to the exterior and whose mistreatment, particularly in carcasses, causes sickness. This leads them to include in their domesticated spaces these bats, gazelles and elephants which governments and experts represent as wild reservoirs. In the different societies we have investigated, zoonotic outbreaks thereby reveal contrasting representations of cross-species pathogens.

At each of our three sites, we often found that conservationists hold an intermediary position between people living with animals and governments following biosecurity measures. As the threat of epidemics coming from animals also reveals the threat of extinction of animal species, and as the protection of biodiversity is shown to mitigate the threat of emerging infectious diseases [37], local knowledge is increasingly connected to a politics of heritage, aimed at conserving animals while protecting those who live with them. In this context, social representations of pathogens integrate indigenous ontologies within stabilized institutional forms. While zoonotic outbreaks can sometimes lead to opposition between indigenous people and conservation experts, as we have seen in the case of FMD management in Mongolia, they can also open possibilities for collaborations between them.

The Mongolian government has made opportunistic use of this politics of heritage and conservation. It supports the application of nomadic herding for the status of international heritage at UNESCO, to strengthen the contribution of herders in the preservation of biodiversity on the one hand, but also the tourism industry in the countryside, on the other. However, in the eyes of NGO’s who support this application, the Mongolian government does not sufficiently fund the development of veterinary care, nor does it provide herders with an effective vaccination program. If Mongolia obtained the status of international heritage for nomadic herding, many herders say that the government would need to foster measures to protect the herd from the threats of zoonotic outbreaks.

To bypass private veterinarians and the National Veterinary Agency, herders have developed a collaboration with international NGOs specialized in animal health and biodiversity conservation. These NGOs provide free veterinary treatments and medicines, regularly send veterinarians to tour the camps, and help herders to develop a small business (wood, leather, sewing, milk, etc.), while coping with climate and public health crises. The same NGOs criticize the specialization of some herders in cashmere wool production, which gives them more money without providing more work. They also insist that this activity weighs too heavily on the ecosystem, does not optimize the regrowth of vegetation, generates overgrazing that causes climatic disasters (*zud*) (snowstorms that freeze the soil, tire the animals that die of hunger and cold) [3], and causes the emergence of animal diseases such as in Siberia [53]. The protection of nomadic knowledge as a heritage thus appears to be a way to protect herders from zoonoses.

Politics of heritage can also conflict with politics of conservation because they create new opportunities for pathogens to cross species barriers. An example of this tension can be found in the annual elephant festival, held since 2007 in the Province of Sayabouli in Laos. In the country, most of the elephant tourist camps are located in the Luang Prabang area, the ancient royal capital of the country nowadays turned into the most touristic place in Laos. In 2015, a caravan of 20 elephants (*caravan xang*) crossed the northern part of the country for 45 days, in order to raise both local and international awareness to help save the last elephants of Laos. The caravan was organized by the major conservation center of the country. The journey ended in Luang Prabang where it was associated with the 20th anniversary celebration of the city as a cultural heritage site of UNESCO. As part of the official activities of the celebration, an elephant parade including more than 30 pachyderms was organized in the city. While the warning on elephant TB aimed at protecting mahouts from their elephants, the risks of a TB outbreak in elephants was perceived by conservationists and tourist camps as a risk of business collapse; hence, little information was communicated to the general public about the subject. Moreover, the politics of conservation left little space for mahouts and elephant owners, who were folklorized during the elephant caravan, the latter wearing ancient costumes dated from ancient royal times to appear in public. There is an opportunity for such knowledge to be integrated into issues related to public health and protection of the environment. By translating risks of TB into more general concerns for threatened species, programs for the surveillance of the disease would probably make more sense for the mahouts and owners. Through the different forms of valorisation it generates, the heritage process should transform knowledge into a pragmatic tool to conserve animal species [28].

To describe these new relations between natural conservation, cultural heritage, and the ontologies of cross-species transmission, we suggest that attention should be given to the way representations are made and circulated. Here we borrow concepts from the anthropology of art, showing how esthetic representations play on polarities in the transformation of natural beings into cultural commodities [34]. We ask how the transformation of local knowledge into expertise by zoonotic outbreaks integrates local actors in a global network, or inversely excludes them.

The Australian case brings such lessons in the domains of indigenous art and wildlife conservation. Since the 1970s, traditional owners and indigenous rangers have been involved in national parks management, combining western technologies and traditional techniques [18]. Many introduced species (wild pigs, cats, cane toads) were targeted and removed while endangered native species like tree kangaroos, cassowaries or bats were protected. Since 2010, the spectacled flying fox is protected under a National Recovery Plan, which aims at maintaining and restoring the flying fox natural habitat in North Queensland.

One of the traditional indigenous methods still used today is to perform the methodical burning of particular areas of land by rangers, under the guidance of community elders and traditional owners. This technique of controlled burning allows for a natural patchwork of fertile soil, where plants grow back stronger and greener, providing a continual reserve of nutrients for the local fauna, even in semi-desert environments. Over the last 50 years, the practice was abandoned in some parts of central Australia as indigenous people were removed from the territory. During a bat survey in Central Australia in the mid 1990s, a team of scientists was investigating the presence of a local ghost bat (*Macroderma gigas*), living in desert caves and deep overhangs under the guidance of the indigenous owner of the land. Because the practice of burning had been abandoned, bats did not find enough food sources and became extinct. Their disappearance left the local indigenous communities in a state of profound grief, knowing that they could not perform the appropriate tasks necessary to maintain their land [7].

In most Australian indigenous cultures, the balance of the ecosystem directly depends on human activities, both physical and spiritual. While indigenous practices such as burning soils maintain the cycle of fertility, some types of ritual actions were also traditionally performed to ensure that the totemic energy of a place (and associated mythical being) was correctly redirected towards the appropriate species, helping the reproduction process of a specific plant or animal. These rituals have many names, but they have been described in classical ethnography as “increase ceremonies” [38]. They include specific songs, gestures, paintings, and objects to be performed or manipulated in order to unlock the totemic energy of a place and maintain the ecological balance.

While these ceremonies are rarely performed in tropical forest areas today, some of their core elements are still used in the creation of objects for museums and the art market. For instance, in the east of the Cape York area (Aurukun), flying fox totems were reinterpreted by Arthur Koo’ekka Pambegan, the guardian of the Kalben ceremony, for public showing. In this sculpture, the bats took the minimalist form of a dozen 20-cm elongated wooden blocks painted with black and red stripes, hanging between two poles [59]. Because the Kalben story is partly secret, the production was closely controlled and limited to museums and fine art markets. Only a fraction of the Kalben myth was made available to the public, including a part on the regenerative capacity of totemic bats which, according to Pambegan, rose from the dead after they were killed. In the same way that Mongolian herders say that pathogens can “silently live” or “survive” in the ground and rise seasonally in certain locations, Aboriginal ritual experts have a special understanding of health as a system of interdependent agents in specific places.

## Conclusion

Animal diseases are new objects for social anthropology because they raise the fundamental questions of the discipline. Research on zoonoses is not only applied anthropology, where knowledge about culture is used to make public health intervention acceptable, but it raises new questions of what *is* culture, knowledge, health and the human. The perception of a zoonotic risk mixes different views, potentially conflicting, of domestic animals and of the threats they introduced from a wild reservoir. The distinction between domestic and wild, or body and soul, is blurred and sometimes reversed when seen from the perspectives of those who live in daily contact with animals.

In this article, we have considered the concept of species barriers as a new starting point for enquiries about problems arising in the domestication of animals. Rather than criticizing the concept of species barrier as a concern for security projected on our relations to nature, we consider different representations of species barriers as ways of bringing new data about human–animal relationships. When pathogens cross species barriers, they enroll a whole range of other invisible beings who take part in the perception of the environment. Asking how these beings can become signs of future threats for humans, we have traced a map of the different boundaries they have to cross.

We have thus shown that social representations help to stabilize zoonotic outbreaks, because humans are attentive to signs of disease based on the knowledge of their environments, in ways that are sometimes compatible and sometimes contradictory with biosecurity interventions. These social representations can be extended and institutionalized by discourses of natural conservation, which may enter into conflict or collaboration with politics of cultural heritage. Because they are reverse processes (transmissions of pathogens from animals to humans and from humans to animals), zoonoses produce complex relations between humans and animals that cannot be categorized by the opposition between natural reservoirs and cultural interventions, but should be analyzed in an ontological process of social representation. What a zoonotic pathogen is and how it should be managed properly are troubling questions open to negotiations between groups, in the management of public health, and between species, in the process of co-evolution.
